# Efficacy and safety of *Si-Jun-Zi-Tang-*based therapies for functional (non-ulcer) dyspepsia: a meta-analysis of randomized controlled trials

**DOI:** 10.1186/s12906-020-03176-z

**Published:** 2021-01-06

**Authors:** Yaping Wang, Bin Liu, Xiuqiong Fu, Tiejun Tong, Zhiling Yu

**Affiliations:** 1 School of Chinese Medicine, Hong Kong Baptist University, Hong Kong, China; 2 Guangzhou Institute of Cardiovascular Disease, the Second Affiliated Hospital of Guangzhou Medical University, Guangzhou, China; 3 Department of Mathematics, Hong Kong Baptist University, Hong Kong, China; 4 Consun Chinese Medicines Research Centre for Renal Diseases, School of Chinese Medicine, Hong Kong Baptist University, Kowloon Tong, Hong Kong, China

**Keywords:** *Si-Jun-Zi-tang*, Traditional Chinese medicine, Functional (non-ulcer) dyspepsia, Meta-analysis, Efficacy, Safety

## Abstract

**Background:**

The traditional Chinese medicine formula *Si-Jun-Zi-Tang* (SJZT) has a long history of application in the treatment of functional dyspepsia (non-ulcer dyspepsia, FD)-like symptoms. SJZT-based therapies have been claimed to be beneficial in managing FD. This study aimed to assess the efficacy and safety of SJZT-based therapies in treating FD by meta-analysis.

**Methods:**

Systematic searches for RCTs were conducted in seven databases (up to February 2019) without language restrictions. Data were analyzed using Cochrane RevMan software version 5.3.0 and Stata software version 13.1, and reported as relative risk (RR) or odds ratio (OR) with 95% confidence intervals (CIs). The primary outcome was response rate and the secondary outcomes were gastric emptying, quality of life, adverse effects and relapse rate. The quality of evidence was evaluated according to criteria from the Cochrane risk of bias.

**Results:**

A total of 341 potentially relevant publications were identified, and 12 RCTs were eligible for inclusion. For the response rate, there was a statically significant benefit in favor of SJZT-based therapies (RR = 1.23; 95% CI 1.17 to 1.30). However, the benefit was limited to modified SJZT (MSJZT). The relapse rate of FD patients received SJZT-based therapies was lower than that of patients who received conventional medicines (OR = 0.23; 95% CI 0.10 to 0.51). No SJZT-based therapies-related adverse effect was reported.

**Conclusion:**

SJZT-based prescriptions may be effective in treating FD and no serious side-effects were identified, but the effect on response rate appeared to be limited to MSJZT. The results should be interpreted with caution as all the included studies were considered at a high risk of bias. Standardized, large-scale and strictly designed RCTs are needed to further validate the benefits of SJZT-based therapies for FD management.

**Trial registration:**

Systematic review registration: [PROSPERO registration: CRD42019139136].

## Background

Functional (non-ulcer) dyspepsia (FD) is one of the most common chronic functional gastrointestinal disorders. According to the Rome IV criteria, FD is characterized by the presence of one or more symptoms, such as postprandial fullness, early satiation, epigastric pain and epigastric burning, none of which can be explained by an organic disease [1]. It affects up to 10–20% of the general population [2, 3]; and most FD patients suffer from a relapsing-remitting course [4–6]. FD significantly reduces patients’ quality of life. The pathogenesis of FD is not fully understood [7]. Many therapies have been proposed for FD, including *Helicobacter pylorieradication* therapy [8], acid-suppression therapy [9], prokinetic agents [10], antidepressants [11], psychological therapy [12], and placebo [10]. However, these therapies are unsatisfactory in efficacy and some of them have serious side effects [13–16].

Nowadays, there is an increasing interest in using complementary and alternative medicine, especially traditional Chinese medicine (TCM), for the management of FD [17–19]. *Si-Jun-Zi-Tang* (SJZT), a well-known TCM formula, has long been used in treating FD in China and Japan [20–22]. It is called “Shikunshito” in Japanese, and “Sagoonjatang, Sagunjatang, Sakoonjatang, Sakunjatang” in Korean. SJZT was first documented in the earliest TCM formula book *Tai-Ping-Hui-Min-He-Ji-Ju-Fang* [23]. The formula contains four herbs: Ginseng Radix et Rhizoma (the dried root and rhizome of *Panax ginseng* C. A. Mey.), Atractylodis Macrocephalae Rhizoma (the dried rhizome of *Atractylodes macrocephala* Koidz.), Poria [the dried sclerotium of *Poria cocos* (Schw.) Wolf] and Glycyrrhizae Radix et Rhizoma (the dried root and rhizome of *Glycyrrhiza uralensis* Fisch., *Glycyrrhiza inflata* Bat. or *Glycyrrhiza glabra* L.). Modern pharmacological studies have demonstrated that SJZT protects the gastric mucosa, improves gastrointestinal motility and immune function of the intestinal mucosa, and balances gut microecology [24, 25]. A considerable number of clinical trials have been conducted to assess the efficacy and safety of SJZT-based therapies in patients with FD. Here, we performed a meta-analysis of randomized controlled trials (RCTs) of SJZT-based therapies in treating FD.

## Methods

### Protocol and registration

The protocol of this study was registered on PERSPERO (CRD42019139136).

### Search strategy

A comprehensive search was carried out in seven electronic databases, including the Cochrane Library, Embase, Medline, Chinese Biomedical Database (CBM), Wanfang, China Science and Technology Journal Database (VIP), and China National Knowledge Infrastructure (CNKI). No publication date or publication status restriction was imposed. Detailed search strategies used in Cochrane Library, Embase and Medline databases are presented in Table 1. We used the Chinese words *四君子汤* (*Si-Jun-Zi-Tang*) and *功能性消化不良* (functional dyspepsia) for the search in CBM, Wanfang, VIP and CNKI. Classic formulas derived from SJZT, e.g. *Liu-Jun-Zi-Tang*, were not involved in this study.

**Table 1:** Search strategy

Database	Period of search	Search strategy
The Cochrane Library	Feb. 2019	1. MeSH descriptor: [dyspepsia] explode all trees 2. dyspepsia or dyspeptic or NUD or FD: ti, ab, kw (Word variations have been searched) 3. indigestion or indigestive: ti, ab, kw (Word variations have been searched) 4. 1 or 2 or 3 5. Sijunzi*: ti, ab, kw (Word variations have been searched) 6. Si-jun-zi*: ti, ab, kw (Word variations have been searched) 7. Shikunshito*: ti, ab, kw (Word variations have been searched) 8. Sa?oonja*: ti, ab, kw (Word variations have been searched) 9. Sa?unja*: ti, ab, kw (Word variations have been searched) 10. 5 or 6 or 7 or 8 or 9 11. 4 and 10
Medline (OvidSP)	1987 to Feb. 2019	1. exp. dyspepsia/ 2. (dyspepsia or dyspeptic or NUD or FD).mp. 3. (indigestion or indigestive).tw. 4. 1 or 2 or 3 5. Sijunzi*.mp. 6. Si-jun-zi*.mp. 7. Shikunshito*.mp. 8. Sa?oonja*.mp. 9. Sa?unja*.mp. 10. 5 or 6 or 7 or 8 or 9 11. 4 and 10
Embase (OvidSP)	1987 to Feb. 2019	1. exp. dyspepsia/ 2. (dyspepsia or dyspeptic or NUD or FD).mp. 3. (indigestion or indigestive).tw. 4. 1 or 2 or 3 5. Sijunzi*.mp. 6. Si-jun-zi*.mp. 7. Shikunshito*.mp. 8. Sa?oonja*.mp. 9. Sa?unja*.mp. 10. 5 or 6 or 7 or 8 or 9 11. 4 and 10

*MeSH* Medical Subject Headings, *NUD* non-ulcer dyspepsia, *FD* functional dyspepsia, *ti* tittle, *ab* abstract; *kw* keyword, mp. the default multi-purpose set of fields, *tw*. text word, *exp.* explosion

### Inclusion criteria

Studies were eligible for inclusion if they met all of the following five criteria: (1) patients were diagnosed with FD either by a clinician or according to specific diagnostic criteria: Rome I II, III or IV criteria [26–29]; (2) studies were conducted as RCTs; (3) effects of SJZT or modified SJZT (MSJZT) in treating FD were assessed; (4) the possible comparisons were as follows: SJZT or modified SJZT (MSJZT) vs. placebo, SJZT or MSJZT plus conventional medicines vs. conventional medicines, SJZT or MSJZT plus conventional medicines vs. placebo; (5) efficacy evaluation criteria were sufficiently described; and (6) treatment lasted for at least 4 weeks.

### Exclusion criteria

Studies were excluded if they met any of the following criteria: (1) patients were diagnosed with diabetes or severe disease in the liver, gallbladder, or the cardiovascular system; (2) patients could not localize their discomfort; (3) pregnant or lactating women were involved; (4) patients were diagnosed with severe depression; (5) SJZT or MSJZT was combined with other Chinese herbal decoctions or with other traditional therapies such as acupuncture; and (6) patients had previously undergone abdominal surgery.

### Data extraction and risk of bias assessment

To avoid bias, two persons (Yaping Wang and Xiuqiong Fu) independently extracted the data and assessed the quality of the involved studies. Disagreements were resolved by a third person (Bin Liu). Extracted characteristics of reports included the first author, year of publication, patients’ basic information, interventions, duration of therapy, outcomes and adverse events.

The methodological quality of each study was assessed according to criteria from the Cochrane risk of bias, including random sequence generation (selection bias), allocation concealment (selection bias), blinding of participants and personnel (performance bias), blinding of outcome assessment (detection bias), incomplete outcome data (attrition bias), selective reporting (reporting bias) and other bias [30].

### Data analysis

Dichotomous data were presented as relative risk (RR) or odds ratio (OR) with 95% confidence intervals (CIs) based on whether the SJZT-based therapies increase or reduce the chance of events [30]. The chi-square test was used to evaluate the heterogeneity and *I*^2^ was used to assess the inconsistency across studies. Values of *I*^2^ ranged from 0 to 100% (*I*^2^ < 40%, might not be important; 30% < *I*^2^ < 60%, moderate heterogeneity; 50% < *I*^2^ < 90%, substantial heterogeneity; 75% < *I*^2^ < 100%, considerable heterogeneity) [30]. The fixed-effect model was used to pool estimates. Potential sources of heterogeneity were identified by sensitivity analysis, subgroup analysis and meta-regression analysis. The covariates in the regression analysis included the intervention of the trial group and the intervention of control group. Potential publication bias was assessed graphically with a funnel plot. The analyses were conducted using the Cochrane RevMan software (version 5.3.0; Cochrane Collaboration, Oxford, UK) and the Stata software (version 13.1; College Station, TX, USA).

## Results

### Study selection

**Fig. 1:**
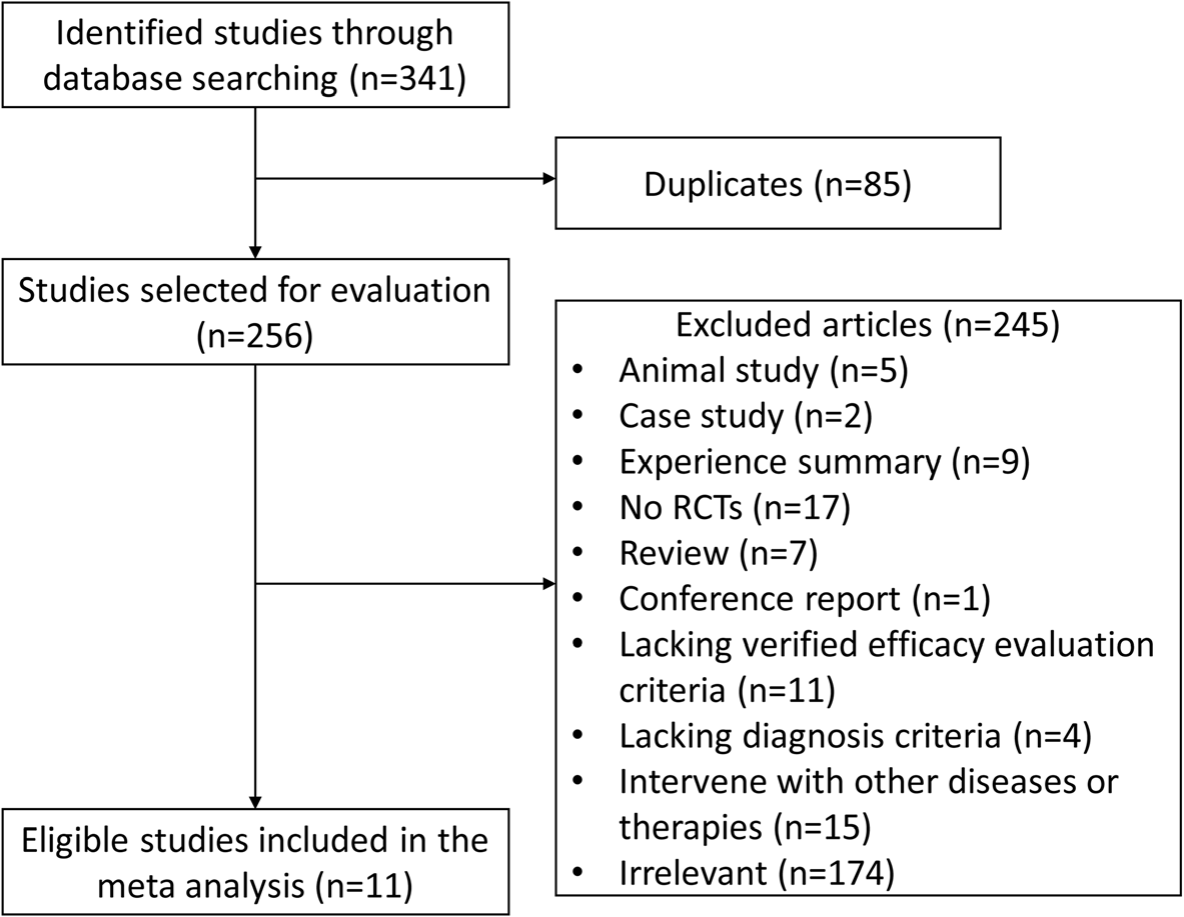
Flow chart of selection process of studies identified in the meta-analysis

A total of 341 records from 7 databases were identified by the search strategy, of which 85 records were duplicates. Of the remaining 256 articles, there were 5 animal studies, 2 case studies, 9 experience summaries, 17 non-RCTs, 7 review articles, 1 conference report, 11 studies that lack sufficient efficacy evaluation criteria, 4 studies that lack diagnosis criteria, 15 studies that involve other diseases or therapies such as acupuncture, and 174 irrelevant articles. Finally, 11 studies, in which there are 12 RCTs {one of the articles reported 2 RCTs according to the different dosage of MSJZT applications: a high dosage (HD), and a low dosage (LD) [31]}, met the inclusion criteria for this meta-analysis (Fig. 1).

### Description of studies

In total, 1241 patients with FD were involved in the 12 separate RCTs, 715 cases in the trial groups and 668 cases in the control groups. There was no significant difference in terms of sample size, age and sex ratio between trial and control groups (Table 2). All of the included 12 trials were conducted in China. SJZT or MSJZT was applied alone or in combination with conventional medicines in the trial groups, whereas conventional medicines or placebos were used in the control groups.

**Table 2:** Characteristics of included randomized controlled trials

Author (Ref.)	Diagnostic criteria	Sample size		Age range, mean (years)		Sex (Male/female)	
		Trial group	Control group	Trial group	Control group	Trial group	Control group
Hu (2001) [32]	Clinical diagnosis and negative investigations	49	36	18–70, 43.5	19.5–69, 42.5	19/30	14/22
Cao (2008) [33]	Clinical diagnosis	49	36	18–70, 43.5	19–69, 42.5	19/30	14/22
Li (2008) [34]	Rome II criteria, TCM diagnostic criteria and negative investigations	45	45	23–68, 39.7	21–65, 43.4	12/33	15/30
Deng and Su (2010)^a^ [31]	Rome III criteria	142	142	19–73, 45.8	19–69, 44.9	50/92	53/89
Deng and Su (2010)^b^ [31]	Rome III criteria	140	142	18–75, 46.7	19–69, 44.9	51/89	53/89
Mu, (2012) [35]	Rome III criteria and negative investigations	63	62	18–60, 34.0	18–60, 35.0	28/35	29/33
Zhang, (2013) [36]	Rome III criteria and negative investigations	60	60	NM, 51.5	NM, 52.8	32/28	35/25
Lv, (2014) [37]	Rome III criteria, TCM diagnostic criteria and negative investigations	41	20	20–68, 47.2	20–68, 45.5	17/24	9/11
Zhang, (2014) [38]	Rome III criteria, TCM diagnostic criteria and negative investigations	29	28	29–63, 50.1	25–64, 47.4	14/15	14/14
Lan and Yuan, (2016) [39]	Clinical diagnosis	33	33	22–69, 50.5	21–69, 49.2	18/15	17/16
Liu et al., (2016) [40]	Rome III criteria, TCM diagnostic criteria and negative investigations	30	30	NM, 45.8	NM, 44.2	13/17	14/16
Li, (2016) [41]	Rome III criteria	34	34	18–59, 37.2	22–58, 37.6	15/19	16/18

*NM* not mentioned

^a^ high dosage; ^b^ low dosage

Patients in 3 trials were prescribed with SJZT [34–36], and patients in the other 9 trials were prescribed with MSJZT [31–33, 37–41]. The detailed herbal compositions of SJZT or MSJZT used in the 12 trials are shown in Table 3. Due to the similar pharmacological activities such as anti-fatigue and immunomodulatory properties, Codonopsis Radix is often used as a cheap substitute for Ginseng Radix et Rhizoma [42, 43]. SJZT or MSJZT was used in combination with conventional medicines in 3 trials [35, 36, 40]. Prokinetic agents, such as domperidone, cisapride or mosapride, were usually used in the control group. Intervention periods ranged from 4 weeks to 60 days. Only one study reported adverse events in the control group. Three trials reported a follow-up period from 2 months to 6 months. Two trials assessed the quality of life and 2 trials measured gastric emptying time (Table 4).

**Table 3:** Compositions of SJZT and MSJZT used in included clinical trials

Study ID	Formula	Compositions of formulas	
		Main herbs	Case-dependently included herbs
Hu (2001) [32]	MSJZT	Codonopsis Radix 10 g, Atractylodis Macrocephalae Rhizoma 12 g, Poria 15 g, Glycyrrhizae Radix et Rhizoma 6 g	Aurantii Fructus Immaturus 15 g, Aucklandiae Radix 10 g, Pinelliae Rhizoma Praeparatum 12 g, Citri Reticulatae Pericarpium 12 g, Coptidis Rhizoma 6 g, Bupleuri Radix 9 g, Citri Sarcodactylis Fructus 10 g, Zingiberis Rhizoma Recens 9 g, Setariae Fructus Germinatus 15 g, Hordei Fructus Germinatus 15 g
Cao (2008) [33]	MSJZT	Codonopsis Radix 10 g, Atractylodis Macrocephalae Rhizoma 12 g, Poria 15 g, Glycyrrhizae Radix et Rhizoma 6 g	Aurantii Fructus Immaturus 15 g, Aucklandiae Radix 10 g, Pinelliae Rhizoma Praeparatum 12 g, Citri Reticulatae Pericarpium 12 g, Coptidis Rhizoma 6 g, Bupleuri Radix 9 g, Citri Sarcodactylis Fructus 10 g, Zingiberis Rhizoma Recens 9 g, Setariae Fructus Germinatus 15 g, Hordei Fructus Germinatus 15 g
Li (2008) [34]	SJZT	Codonopsis Radix 15 g, Atractylodis Macrocephalae Rhizoma 10 g, Poria 30 g, Glycyrrhizae Radix et Rhizoma 5 g	No
Deng and Su (2010)^a^ [31]	MSJZT	Codonopsis Radix 18 g, Atractylodis Macrocephalae Rhizoma 18 g, Poria 18 g, Glycyrrhizae Radix et Rhizoma 10 g	Aurantii Fructus Immaturus 10 g, Magnoliae Officinalis Cortex 10 g, Aucklandiae Radix 10 g, Amomi Fructus 10 g, Alpiniae Officinarum Rhizoma 10 g, Galli Gigerii Endothelium Corneum 20 g, Crataegi Fructus 15 g, Hordei Fructus Germinatus 15 g, Massa Medicata Fermentata 15 g
Deng and Su (2010)^b^ [31]	MSJZT	Codonopsis Radix 10 g, Atractylodis Macrocephalae Rhizoma 10 g, Poria 10 g, Glycyrrhizae Radix et Rhizoma 10 g	Aurantii Fructus Immaturus 10 g, Magnoliae Officinalis Cortex 10 g, Aucklandiae Radix 10 g, Amomi Fructus 10 g, Alpiniae Officinarum Rhizoma 10 g, Galli Gigerii Endothelium Corneum 20 g, Crataegi Fructus 15 g, Hordei Fructus Germinatus 15 g, Massa Medicata Fermentata 15 g
Mu, (2012) [35]	SJZT	Codonopsis Radix 15 g, Atractylodis Macrocephalae Rhizoma 10 g, Poria 30 g, Glycyrrhizae Radix et Rhizoma 5 g	No
Zhang, (2013) [36]	SJZT	Codonopsis Radix 15 g, Atractylodis Macrocephalae Rhizoma 10 g, Poria 30 g, Glycyrrhizae Radix et Rhizoma 5 g	No
Lv, (2014) [37]	MSJZT	Codonopsis Radix 15 g, Atractylodis Macrocephalae Rhizoma 15 g, Poria 15 g, Glycyrrhizae Radix et Rhizoma 5 g	Amomi Fructus 8 g, Aurantii Fructus Immaturus 15 g
Zhang, (2014) [38]	MSJZT	Codonopsis Radix 15 g, Atractylodis Macrocephalae Rhizoma 15 g, Poria 15 g, Glycyrrhizae Radix et Rhizoma 3 g	Amomi Fructus 8 g, Aurantii Fructus Immaturus 15 g
Lan and Yuan, (2016) [39]	MSJZT	Codonopsis Radix 10 g, Atractylodis Macrocephalae Rhizoma 12 g, Poria 15 g, Glycyrrhizae Radix et Rhizoma 6 g	Setariae Fructus Germinatus 15 g, Hordei Fructus Germinatus 15 g, Aurantii Fructus Immaturus 15 g, Pinelliae Rhizoma Praeparatum 12 g, Citri Reticulatae Pericarpium 12 g, Citri Sarcodactylis Fructus 10 g, Aucklandiae Radix 10 g, Zingiberis Rhizoma Recens 9 g, Bupleuri Radix 9 g, Coptidis Rhizoma 6 g
Liu et al., (2016) [40]	MSJZT	Ginseng Radix et Rhizoma 15 g, Atractylodis Macrocephalae Rhizoma 15 g, Poria 15 g, Glycyrrhizae Radix et Rhizoma 10 g	Aucklandiae Radix 10 g, Amomi Fructus 10 g
Li, (2016) [41]	MSJZT	Codonopsis Radix 15 g, Atractylodis Macrocephalae Rhizoma 15 g, Poria 15 g, Glycyrrhizae Radix et Rhizoma 10 g	Citri Reticulatae Pericarpium 10 g, Aucklandiae Radix 10 g, Bupleuri Radix 15 g, Aurantii Fructus 15 g, Raphani Semen 20 g, Paeoniae Radix Alba 20 g, Citri Sarcodactylis Fructus 20 g

^a^ high dosage; ^b^ low dosage

**Table 4:** Interventions and outcomes of included trials

Study ID	Intervention		Duration/ follow up	Outcome measures	Adverse event
	Trial group	Control group			
Hu (2001) [32]	MSJZT	Pantoprazole	4 weeks/ NM	1) Response rate 2) Gastric emptying	NM
Cao (2008) [33]	MSJZT	Pantoprazole	4 weeks/ NM	Response rate	NM
Li (2008) [34]	SJZT	Pantoprazole	4 weeks/ 2 months	Response rate	Control group: 8 cases with abdominal discomfort, bloating, diarrhea
Deng and Su (2010)^a^ [31]	MSJZT (High dose)	Pantoprazole	4 weeks/ NM	Response rate	NM
Deng and Su (2010)^b^ [31]	MSJZT (low dose)	Pantoprazole	4 weeks/ NM	Response rate	NM
Mu (2012) [35]	SJZT + Domperidone + Omeprazole	Domperidone + Omeprazole	4 weeks/ NM	Response rate	NO
Zhang (2013) [36]	SJZT + Mosapride Citrate	Mosapride Citrate	4 weeks/ 6 months	1) Response rate 2) Relapse rate	NM
Lv (2014) [37]	MSJZT	Placebo	4 weeks	1) Quality of life 2) Response rate 3) Gastric emptying	NO
Zhang (2014) [38]	MSJZT	Placebo	4 weeks/ NM	1) Quality of life 2) Response rate	NO
Lan and Yuan (2016) [39]	MSJZT	Pantoprazole	4 weeks/ NM	Response rate	NM
Liu et al. (2016) [40]	MSJZT + Domperidone	Domperidone	8 weeks/ NM	Response rate	NO
Li (2016) [41]	MSJZT	Domperidone	60 days/ 6 months	1) Response rate 2) Relapse rate	NM

*NM* not mentioned

^a^ high dosage; ^b^ low dosage

### Methodological quality

**Fig. 2:**
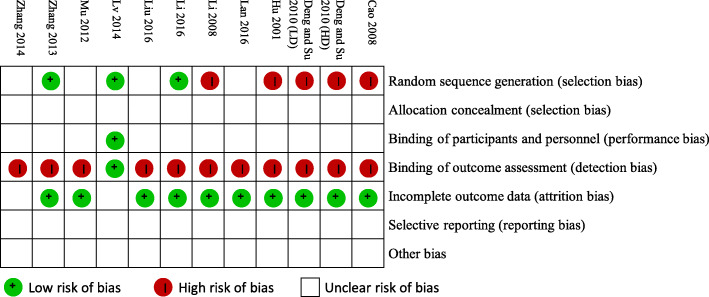
Risk of bias summary

The risk of bias was generally high in all of the included trials (Fig. 2). All 12 trials were mentioned as ‘randomized’. Among them, only 3 trials specified the method of randomization (random number table) [36, 37, 41], and 5 trials had an inappropriate randomization method [31–34]. None of them described the allocation concealment procedure. Only one trial was double-blinded and had a placebo control [37]. Two trials mentioned dropout rates, without giving sufficient reasons for each dropout, so the attrition biases of these 2 trials were unclear [37, 38]. None of these trials provided a research protocol, and they were all rated as having an unclear risk of bias in selective reporting of outcomes. All of the 12 trials were also rated as having an unclear risk of other bias.

### Outcome measures

The primary outcome measure was the response rate, and the secondary outcome measures were gastric emptying, quality of life, adverse effects, and relapse rate.

#### Response rate

**Fig. 3:**
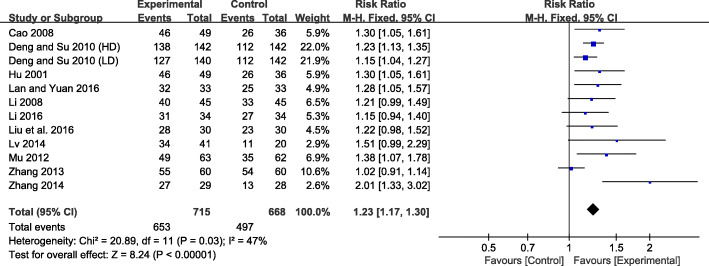
Forest plot for the response rate of SJZT or MSJZT in patients with FD

**Fig. 4:**
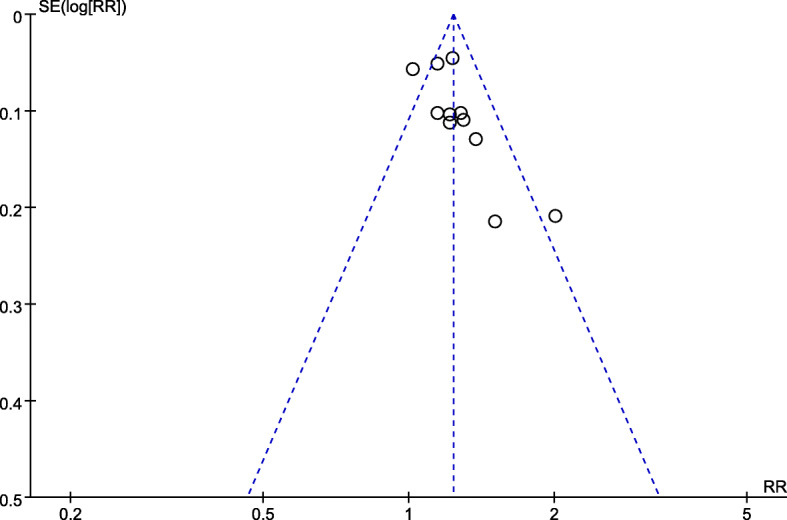
Funnel plot of included trials for response rate

The response rate was defined as the proportion of patients experiencing ‘complete recovery’ and ‘partial improvement’ on global FD symptoms during intervention periods. The pooled RR for response rate was 1.23 (95% CI 1.17 to 1.30), with a moderate heterogeneity across studies (*I*^*2*^ = 47%, *P* = 0.03) (Fig. 3). There was an obvious asymmetry in the funnel plot, indicating the existence of publication bias (Fig. 4). A sensitivity analysis was performed to investigate potential sources of heterogeneity (Table 5). After removing the study with the smallest number of enrolled patients [38], the heterogeneity moderately decreased. After removing the study with the highest average age of FD patients [36], the heterogeneity reduced significantly. The overall response rate slightly altered after excluding one of the two studies. Therefore, the difference in the population size and average age of FD patients may account for the heterogeneity across these trials.

**Table 5:** Relative risks (RRs) and heterogeneity tests for sensitivity analyses

Excluded study	Pooled RR (95% CI)	*P*_heterogeneity_; *I*^2^
Hu (2001) [32]	1.22 (1.13, 1.32)	0.03; 51%
Cao (2008) [33]	1.22 (1.13, 1.32)	0.03; 51%
Li (2008) [34]	1.23 (1.13, 1.33)	0.02; 52%
Deng and Su (2010)^a^ [31]	1.23 (1.13, 1.34)	0.02; 52%
Deng and Su (2010)^b^ [31]	1.24 (1.14, 1.35)	0.02; 52%
Mu (2012) [35]	1.21 (1.13, 1.31)	0.04; 48%
Zhang (2013) [36]	1.24 (1.17, 1.30)	0.38; 7%
Lv (2014) [37]	1.21 (1.13, 1.31)	0.04; 48%
Zhang (2014) [38]	1.19 (1.13, 1.27)	0.19; 27%
Lan and Yuan (2016) [39]	1.22 (1.13, 1.32)	0.02; 51%
Liu et al. (2016) [40]	1.23 (1.13, 1.33)	0.02; 52%
Li (2016) [41]	1.23 (1.14, 1.33)	0.02; 52%

*RR* relative risk, *CI* confidence interval

^a^ high dosage; ^b^ low dosage

We also conducted subgroup analyses according to the intervention used in trial group and the intervention in control group. These revealed that the beneficial effect of SJZT-based therapies appeared to be limited to MSJZT (Table 6). In addition, after the RCTs were divided into 2 groups based on the intervention in control group (placebo or conventional medicines), the heterogeneity was moderately decreased, suggesting that the intervention in control group is another source for the observed heterogeneity. Results of the meta-regression analysis also showed that the intervention in control group was a significant source of heterogeneity (slope = 0.74; 95% CI 0.10 to 1.37; *P* = 0.03) (Table 7).

**Table 6:** Subgroup analysis of response rate of SJZT or MSJZT in patients with FD

	Subgroups	No. of trials	No. of patients	Pooled RR (95% CI); *P*-value	*P*_heterogeneity_; *I*^2^
All studies		12	1383	1.23 (1.17, 1.30); *P* < 0.00001	0.03; 47%
Intervention in trial group	SJZT	1	90	1.21 (0.99, 1.49); *P* = 0.06	Not applicable
	MSJZT	8	988	1.24 (1.16, 1.34); *P* < 0.00001	0.20; 29%
	SJZT + conventional medicines	2	245	1.17 (0.81, 1.68); *P* = 0.40	0.009; 85%
	MSZT + conventional medicines	1	60	1.22 (0.98, 1.52); *P* = 0.08	Not applicable
Intervention in control group	Placebo	2	118	1.74 (1.30, 2.34); *P* = 0.0002	0.34; 0%
	Conventional medicines	10	1265	1.20 (1.15, 1.26); *P* < 0.00001	0.21; 25%

*RR* relative risk, *CI* confidence interval

**Table 7:** Results of meta-regression analysis for response rate

Covariates	Coefficient	Std.err.	*t*	*P*	95% CI
Intervention in control group	0.74	0.29	2.58	0.03	(0.10, 1.37)
Intervention in trail group					
MSJZT	−0.03	0.57	−0.05	0.96	(−1.35, 1.30)
SJZT + conventional medicines	0.03	0.67	0.04	0.97	(−1.51, 1.57)
MSZT + conventional medicines	0.18	0.80	0.23	0.83	(−1.65, 2.02)

*CI* confidence interval

#### Gastric emptying

Two trials measured gastric emptying time [32, 37]. There existed differences in the method measuring gastric emptying. Therefore, these outcomes cannot be properly assessed and incorporated into results.

#### Quality of life

Quality of life in patients with FD was assessed in 2 trials using the Medical Outcomes Study Short Form 36-Item Health Survey (SF-36) [37, 38]. But the methods for calculation of SF-36 scores were not uniform; thus, these outcomes cannot be properly assessed and incorporated into results.

#### Adverse events

Among the 12 trials, 4 trials reported no adverse events [35, 37, 38, 40] and 7 trials did not mention adverse events [31–33, 36, 39, 41]. One trial reported adverse effects such as abdominal discomfort, bloating and diarrhea in the conventional medicine control group [34].

#### Relapse rate

**Fig. 5:**
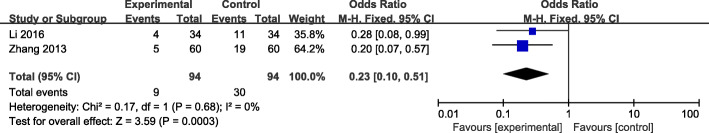
Forest plot of relapse rate

Two trials provided 6-month follow-up data [36, 41]. The relapse rate was significantly lower in the trial group than in the control group. The overall pooled estimate OR of recurrence rate was 0.23 (95% CI 0.10 to 0.51), with low heterogeneity (*I*^*2*^ = 0%, *P* = 0.68) (Fig. 5).

## Discussion

Although the pathophysiology of FD is still unclear, some pathogenic mechanisms have been proposed. Theses mechanisms include duodenal hypersensitivity [44], impaired gastric emptying [45, 46], impaired gastric accommodation [47], *Helicobacter pylori* infection [48] and psychological disorders [49]. Several therapeutic strategies have been proposed accordingly [50], but the efficacy and safety of these strategy-based therapies remain controversial; hence, the need for safe and effective therapeutics for patients with FD remains. For thousands of years, herbal formulas, such as SJZT, have been prescribed for managing FD-like symptoms. Modern studies showed that SJZT exhibits various pharmacological activities such as gastric emptying promotion, gastrointestinal protection, and gastrointestinal tract motility regulation [38, 51–53]. These studies indicate that SJZT-based therapies against FD are promising.

Many researchers have claimed that SJZT-based therapies are effective and safe in managing FD. To assess the reliability of their claims, we conducted a meta-analysis. Based on rigorous methodology and contemporary literature search, 12 eligible RCTs involving 1241 subjects were finally obtained for analysis by two investigators independently. SJZT-based prescriptions were used in trial groups alone or in combination with conventional medicines, whereas conventional medicines or placebo was used in the control groups. Pantoprazole, a prokinetic agent, was one of the most frequently used drugs in control groups. The characteristics and risk of bias of the included trials have been summarized. Publication bias was assessed by funnel plot. In addition, we performed a sensitivity analysis, subgroup analysis and meta-regression analysis to explore possible reasons for heterogeneity across studies.

The meta-analysis results demonstrated that SJZT-based therapies may be effective for treating FD when data from all trials were pooled. However, the beneficial effect on response rate appeared to be limited to MSJZT. Total numbers of relapse events were significantly lower among those using SJZT-based prescriptions. No serious therapy-related adverse event was observed. There was moderate heterogeneity in results with respect to response rates. Differences in population size, the average age of FD patients and the intervention in control group may account for the heterogeneity across studies.

The meta-analysis results have limitations and need to be interpreted properly. First, all the included studies were conducted in China; lack of data from other countries limits the generalizability of the results. Second, MSJZT was used in most trials and the modifications vary among trials. Different modifications may lead to different therapeutic effects. In addition, there were a variety of control interventions that may also increase heterogeneity of the included trials. Third, the sample size of each trial involved in this analysis was not big enough to draw reliable conclusions. Fourth, the diagnostic criteria and end points for defining patients with FD were based on symptoms, which means even a small shift in the criteria when recruiting patients into trials, or small changes in the end points when evaluating the outcomes may alter the outcome of trials [54, 55]. Fifth, all the included studies are of poor methodological quality, and none of these clinical trials published protocols.

## Conclusions

The results of this meta-analysis suggested that, overall, SJZT-based prescriptions are more effective than placebo or conventional treatment for FD management in improving response rate and reducing relapse rate. This work provides modern scientific evidence for the beneficial effects of SJZT-based therapies in treating FD. However, due to the low methodological quality of the included RCTs, the results should be interpreted with caution. Standardized large-scale and strictly designed RCTs that follow relevant guidelines, such as CONSORT for herbal medicines [56], are encouraged to further validate the benefits of SJZT-based therapies for treating FD.

## Data Availability

The datasets supporting the conclusions of this article are included within the article.

## Abbreviations

SJZT: *Si-Jun-Zi-Tang*
FD: Functional dyspepsia
RCTs: Randomized controlled trials
RR: Relative risk
OR: Odds ratio
CIs: Confidence intervals
TCM: Traditional Chinese medicine
CBM: Chinese Biomedical Database
VIP: China Science and Technology Journal Database
CNKI: China National Knowledge Infrastructure
MeSH: Medical Subject Headings
NUD: Non-ulcer dyspepsia
FD: Functional dyspepsia
ti: Tittle
ab: Abstract
kw: Keyword
mp.: The default multi-purpose set of fields
tw.: Text word
exp.: Explosion
MSJZT: Modified *Si-Jun-Zi-Tang*
MD: Mean difference
HD: High dosage
LD: Low dosage
NM: Not mentioned
SF-36: Medical Outcomes Study Short Form 36-Item Health Survey

## References

[1] Stanghellini V, Chan FK, Hasler WL, Malagelada JR, Suzuki H, Tack J (2016). Gastroduodenal disorders. Gastroenterology..

[2] Aro P, Talley NJ, Ronkainen J, Storskrubb T, Vieth M, Johansson SE (2009). Anxiety is associated with uninvestigated and functional dyspepsia (Rome III criteria) in a Swedish population-based study. Gastroenterology..

[3] Kaji M, Fujiwara Y, Shiba M, Kohata Y, Yamagami H, Tanigawa T (2010). Prevalence of overlaps between GERD, FD and IBS and impact on health-related quality of life. J Gastroenterol Hepatol.

[4] Agréus L, Svärdsudd K, Talley NJ, Jones MP, Tibblin G (2001). Natural history of gastroesophageal reflux disease and functional abdominal disorders: a population-based study. Am J Gastro..

[5] Halder SL, Locke GR, Schleck CD, Zinsmeister AR, Melton LJ, Talley NJ (2007). Natural history of functional gastrointestinal disorders: a 12-year longitudinal population-based study. Gastroenterology..

[6] Ford A, Forman D, Bailey A, Axon A, Moayyedi P (2008). Fluctuation of gastrointestinal symptoms in the community: a 10-year longitudinal follow-up study. Aliment Pharm Therap.

[7] Mazzoleni LE, Sander GB, de Magalhães Francesconi CF, Mazzoleni F, Uchoa DM, De Bona LR (2011). Helicobacter pylori eradication in functional dyspepsia: HEROES trial. Arch Intern Med.

[8] Iwakiri R, Tominaga K, Furuta K, Inamori M, Furuta T, Masuyama H (2013). Randomised clinical trial: rabeprazole improves symptoms in patients with functional dyspepsia in Japan. Aliment. Pharm. Therap..

[9] Moayyedi P, Soo S, Deeks J, Delaney B, Innes M, Forman D. Pharmacological interventions for non-ulcer dyspepsia. Cochrane Database Syst Rev. 2006;4:CD001960. 10.1002/14651858.CD001960.pub3 17054151

[10] Talley NJ, Locke GR, Saito YA, Almazar AE, Bouras EP, Howden CW (2015). Effect of amitriptyline and escitalopram on functional dyspepsia: a multicenter, randomized controlled study. Gastroenterology..

[11] Orive M, Barrio I, Orive V, Matellanes B, Padierna J, Cabriada J (2015). A randomized controlled trial of a 10 week group psychotherapeutic treatment added to standard medical treatment in patients with functional dyspepsia. J Psychosom Res.

[12] Lee K-J, Kindt S, Tack J (2004). Pathophysiology of functional dyspepsia. Best Pract Res Cl Gastro.

[13] Bosco D, Plastino M, Marcello MG, Mungari P, Fava A (2009). Acute hemifacial dystonia possibly induced by clebopride. Clin Neuropharmacol.

[14] Shin HW, Chung SJ (2012). Drug-induced parkinsonism. J Clin Neurol.

[15] Quigley EM (2015). Prokinetics in the management of functional gastrointestinal disorders. J Neurogastroenterol Motil.

[16] Leelakanok N, Holcombe A, Schweizer ML (2016). Domperidone and risk of ventricular arrhythmia and cardiac death: a systematic review and meta-analysis. Cl Drug Invest.

[17] Dossett ML, Davis RB, Lembo AJ, Yeh GY (2014). Complementary and alternative medicine use by US adults with gastrointestinal conditions: results from the 2012 National Health Interview Survey. Am J Gastro.

[18] Hung A, Kang N, Bollom A, Wolf JL, Lembo A (2015). Complementary and alternative medicine use is prevalent among patients with gastrointestinal diseases. Digest Dis Sci.

[19] Vlieger AM, Blink M, Tromp E, Benninga MA (2008). Use of complementary and alternative medicine by pediatric patients with functional and organic gastrointestinal diseases: results from a multicenter survey. Pediatrics..

[20] Zhang S, Chen Z, Xu W, Wang HB (2008). Study on distribution characteristic of syndrome of 565 cases of functional dyspepsia by twice differentiation of symptoms and signs based on the ‘cold, heat, deficiency, excess’. Chin J Tradit Chin Med Pharm.

[21] Zhang S, Wang H, Li Q (2010). Chinese consensus on diagnosis and treatment of functional dyspepsia. Chin J Integr Tradit West Med Dig.

[22] Arumugam S, Watanabe K. Japanese kampo medicines for the treatment of common diseases: focus on inflammation: Academic; 2017. P.192.

[23] Liu JY (2007). Taipping Huimin Hejijufang.

[24] Ji YF, Wang RJ, Li XB (2016). Research progress on chemical constituents and pharmacological effects of Sijunzi decoction. Chin Tradit Herb Drugs.

[25] Wang YL, Wang Y (2014). Effects of Sijunzi dripping pill on gastrointestinal motility of mice. Chin Herb Med.

[26] Drossman DA (1994). The functional gastrointestinal disorders: diagnosis, pathophysiology, and treatment: a multinational consenus.

[27] Drossman DA (1999). The functional gastrointestinal disorders and the Rome II process. Gut.

[28] Drossman DA (2006). The functional gastrointestinal disorders and the Rome III process. Gastroenterology..

[29] Drossman DA (2016). Functional gastrointestinal disorders: history, pathophysiology, clinical features, and Rome IV. Gastroenterology..

[30] Higgins JPT, Green S. Cochrane handbook for systematic reviews of interventions 5.1.0. The Cochrane Collaboration. 2011. http://www.cochrane-handbook.org.

[31] Deng YB, Su R (2010). Effective observation on treating functional dyspepsia with different dosage four gentlemen decoction with the card. Clin J Chinese Med.

[32] Hu JF (2001). Clinical observation on Jiawei Sijunzi decoction in treating functional dyspepsia.

[33] Cao JG (2008). Treating 49 cases of functional dystrophy of spleen deficiency and liver stagnation with Jiawei Sijunzi decoction. Chin. J Integr Tradit Western Med Digestion..

[34] Li YH (2008). Clinical observation on treatment of spleen and stomach eeak type functional dyspepsia with Sijunzi decoction. Beijing J Tradit Chin Med.

[35] Mu DC (2012). Effect of Sijunzi decoction in the treatment of functional dyspepsia. Modern Med J Chin.

[36] Zhang ZY (2013). Clinical observation on the treatment of functional dyspepsia with Xiaopi Sanjie method. Chin J Med Guide.

[37] Lv L (2014). Study of the clinical observation and experiments on treating distention and fullness disease (functional dyspepsia) with the invigorating spleen method of traditional Chinese medicine.

[38] Zhang W (2014). Study of the clinical observation and experiments on treating functional dyspepsia (FD) of spleen deficiency with the decoction of Sijunizijiawei.

[39] Lan HB, Yuan HP (2016). Effect of Jiawei Sijunzi decoction on functional dyspepsia of spleen deficiency type. Chin J Urban Rural Enterp Hyg.

[40] Liu HB, Liu FX, Zhang W (2016). Clinical efficacy of Xiangsha Sijunzi decoction combined with domperidone in the treatment of functional dyspepsia with spleen deficiency and qi stagnation. J Med Front.

[41] Li RF (2016). Treatment of 68 cases of functional dyspepsia with sijunzi decoction. Guide Chin Med.

[42] Chen MH, Li YH, Zhou JG, Hu W (2016). Textual research and pharmacological analysis of partly functional replacing Panax Ginseng with Codonopsis Pilosula. J China Three Gorges Univ (Natural Sciences).

[43] Wang Z, Ng T, Yeung H, Xu G (1996). Immunomodulatory effect of a polysaccharide-enriched preparation of Codonopsis pilosula roots. Gen Pharmac.

[44] Tack J, Caenepeel P, Fischler B, Piessevaux H, Janssens J (2001). Symptoms associated with hypersensitivity to gastric distention in functional dyspepsia. Gastroenterology..

[45] Sarnelli G, Caenepeel P, Geypens B, Janssens J, Tack J (2003). Symptoms associated with impaired gastric emptying of solids and liquids in functional dyspepsia. Am J Gastro..

[46] Shindo T, Futagami S, Hiratsuka T, Horie A, Hamamoto T, Ueki N (2009). Comparison of gastric emptying and plasma ghrelin levels in patients with functional dyspepsia and non-erosive reflux disease. Digestion..

[47] Tack J, Piessevaux H, Coulie B, Caenepeel P, Janssens J (1998). Role of impaired gastric accommodation to a meal in functional dyspepsia. Gastroenterology..

[48] Suzuki H, Moayyedi P (2013). Helicobacter pylori infection in functional dyspepsia. Nat Rev Gastro Hep.

[49] Yamawaki H, Futagami S, Shimpuku M, Sato H, Wakabayashi T, Maruki Y (2014). Impact of sleep disorders, quality of life and gastric emptying in distinct subtypes of functional dyspepsia in Japan. J Neurogastro Motil.

[50] Madisch A, Andresen V, Enck P, Labenz J, Frieling T, Schemann M (2018). The diagnosis and treatment of functional dyspepsia. Deutsch Aerztebl Intern.

[51] Zhong ZS, Huang HP, Zhang W, Lin XF, Zhong RM, Ge YH (2017). Disorder in CNP-NPRB-cGMP pathway of gastric antral smooth muscle of functional dyspepsia model rats with spleen-deficiency syndrome and intervening mechanism of Sijunzi tang. Chin J Exp Tradit Med Formulae.

[52] Song HP, Li RY, Wei YX, Li X, Yu HH, Yuan ZY (2016). Effect of Sijunzi tang in promoting migration and proliferation of small intestinal epithelial cells by up-regulating c-Myc expression. Chin J Exp Tradit Med Formulae.

[53] Tu XH (2016). Studies on the effects of Sijunzi decoction polysaccharide on polyamines-mediated calcium signaling pathway during intestinal epithelial cell migration.

[54] Holtmann G, Talley NJ, Liebregts T, Adam B, Parow C (2006). A placebo-controlled trial of itopride in functional dyspepsia. New Engl J Med.

[55] Vakil NB, Howden CW, Moayyedi P, Tack J. White Paper AGA: Functional dyspepsia. Clin Gastroenterol Hepatol 2017;8:1191–1194. 10.1016/j.cgh.2017.05.013 28529164

[56] Cheng CW, Wu TX, Shang HC, Li YP, Altman DG, Moher D (2017). CONSORT extension for Chinese herbal medicine formulas 2017: recommendations, explanation, and elaboration. Ann Intern Med.

